# Using a Cellular System to Directly Assess the Effects of Cosmetic Microemulsion Encapsulated DeoxyArbutin

**DOI:** 10.3390/ijms222313110

**Published:** 2021-12-03

**Authors:** Nai-Fang Chang, Feng-Jie Tsai, Ya-Min Zheng, Wei-Hsiang Huang, Chih-Chien Lin

**Affiliations:** 1 Department of Cosmetic Science, Providence University, 200, Sec. 7, Taiwan Boulevard, Shalu Dist., Taichung 43301, Taiwan; nfchang@pu.edu.tw (N.-F.C.); tftsay@pu.edu.tw (F.-J.T.); g1070048@gm.pu.edu.tw (Y.-M.Z.); wesley.huang.tw@gmail.com (W.-H.H.); 2 Guidance Association of Taiwan Aromaplants (GATA), 1F., No. 132-9, Tianmu W. Rd., Beitou Dist., Taipei City 112, Taiwan

**Keywords:** deoxyArbutin, microemulsion, polysorbate, surfactants, cell-based assay, cosmetics

## Abstract

DeoxyArbutin (dA) is a tyrosinase inhibitor that has effective skin-lightening activity and has no obvious cytotoxicity toward melanocytes. With the aim of directly evaluating the effects of microemulsions containing dA on cells, we developed oil-in-water (O/W) microemulsions with relatively lower cytotoxicities by using polysorbate-series surfactants. Measurement of the transparent properties and particle size analysis at different storage time periods revealed that the developed microemulsions were stable. Moreover, the developed microemulsions had direct effects on B16-F10 mouse melanoma cells. The anti-melanogenesis activities of dA-containing microemulsions were evidently better than that of the free dA group. The results demonstrated that the developed microemulsion encapsulating dA may allow the use of deoxyArbutin instead of hydroquinone to treat dermal hyperpigmentation disorders in the future.

## 1. Introduction

**Figure 1:**
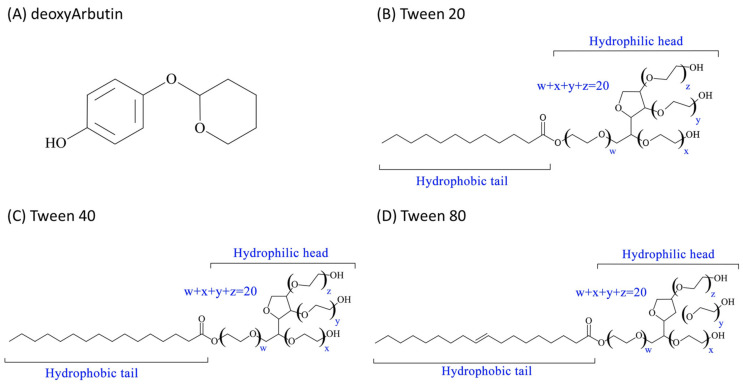
Chemical structures of the compounds and surfactants used: (**A**) deoxyArbutin; (**B**) Tween 20; (**C**) Tween 40; (**D**) Tween 80.

DeoxyArbutin (4-[(tetrahydro-2H-pyran-2-yl) oxy] phenol; dA) is a molecule modified from arbutin by Dr. Boissy’s research group (Figure 1A). This reversible tyrosinase inhibitor has effective skin-lightening activity and shows no obvious cytotoxicity against melanosomes on melanocytes in vivo [1,2]. Therefore, dA has strong skin-lightening and antioxidant activity. However, our earlier studies also found that the molecule is unstable and may decay into hydroquinone (HQ) in high-energy environments induced by treatment with light or heat [3,4,5]. Hence, the formulations used, so that dA can maintain its stability and efficacy, are very important. For this reason, the dA market is still restricted, and only a few cosmetic products can be found in some countries such as the Philippines and USA. Moreover, the Scientific Committee on Consumer Safety (SCCS) of the European Union (EU) banned dA in cosmetics due to safety concerns on 5 July 2021. The use of 3% dA in face creams cannot be considered non-toxic (opinion No. SCCS/1554/15) because dA releases HQ [6]. Therefore, taking advantage of dA instead of HQ to treat hyperpigmentation disorders of the skin may be a good way to utilize this effective skin-lightening agent [7].

A microemulsion is a type of emulsion that is a transparent, thermodynamically stable and homogeneous mixture of an oil phase, a water phase, surfactants and co-surfactants. This emulsion type can be spontaneously formulated without external forces [8,9]. The diameter of most dispersed droplets of a microemulsion range from 1 to 100 nm. Compared with a normal emulsion (macroemulsion), a microemulsion has smaller micelles and better stability [10]. Because this emulsion system achieves minimum free energy and displays no trend of separating into the different phases, microemulsions have many excellent properties for applications in the cosmetic, food and pharmaceutical fields [11,12,13]. The cosmetic industry is regularly looking for innovative products that will combine both recognized biological functions and effective delivery systems. Therefore, microemulsion formulation is promising for the development of the cosmetic industry [14]. However, the majority of testing methods for microemulsions are performed in animal studies or human trials due to the emulsion systems being applied to skin tissue [15]. On the other hand, cell-based experiments are often only used to assess the activity or cytotoxicity of individual components. However, the functions of an emulsion are achieved by all their constituents and even by the structures [9]. Therefore, if a cell-based system was used to measure the effects of a microemulsion directly, it would have the advantage of allowing direct analysis of the developed microemulsion samples, regarding both their activities and cytotoxicities prior to animal studies or human trials.

The polysorbate series of surfactants (polyethylene glycosylated (PEGylated) sorbitan fatty acid esters; Tween surfactants (Figure 1)) are the most frequently used non-ionic surfactants in the delivery formulation of microemulsion-based functional compounds. Typically, the polysorbate series of surfactants are used in pharmaceuticals to improve the solubility and bioavailability of drugs [16]. Many studies have shown that Tween surfactants are a very convenient and safe choice for utilization as the excipient for microemulsion formulations [17,18]. In this study, to achieve the purpose of evaluating the effects of a microemulsion on cells, we chose a non-ionic polysorbate series of surfactants (Tween 20, 40 and 80) and a co-surfactant polyethylene glycol 200 (PEG 200), which have a relatively low cytotoxicity [19], for the formulation of oil-in-water (O/W) microemulsions. The chemical structures of the Tween 20, 40 and 80 surfactants are shown in Figure 1. The developed microemulsions encapsulating deoxyArbutin (ME-dA) were tested for their transparent properties and particle size after different storage periods. Consequently, the effects of the diluted microemulsion samples were directly assessed on B16-F10 melanoma cells to confirm their cytotoxicity and whitening activity. 

## 2. Results and Discussion

### 2.1. Formulation of the Microemulsion Encapsulating dA

To develop safe microemulsions for cellular assessment, we chose the polysorbate series of surfactants, including Tween 20, 40 and 80 and the co-surfactant PEG 200 as the surfactant components. Furthermore, a common and safe synthetic oil, isopropyl palmitate (IPP), was used as the oil phase in the microemulsions [20]. Moreover, IPP exhibits good solubility for dA, which could provide a favorable environment for the stability and biocompatibility of dA. The components of the microemulsion formulations are listed in Table 1. The deoxyArbutin-containing microemulsions formulated from Tween 20, Tween 40 and Tween 80 were named ME-20-dA, ME-40-dA and ME-80-dA, respectively. Because the developed microemulsions were O/W-type microemulsions, to encapsulate the dA inside the droplets, the IPP oil phase was fixed at a lower level, around 2%. Additionally, the Tween series surfactants combined with co-surfactants (surfactant/co-surfactant mixture) composed over 25%, and the Tween series surfactants had a suitable ratio with the co-surfactants of around 4:1 [21]. This suitable ratio was determined by the function of the co-surfactant, which should fill a vacancy between surfactant and stabilize the interfacial tension [22]. Moreover, high surfactant-content microemulsion formulations with over 20–25% Tween surfactants that have already been used in industrial settings [16]. 

**Figure 2:**
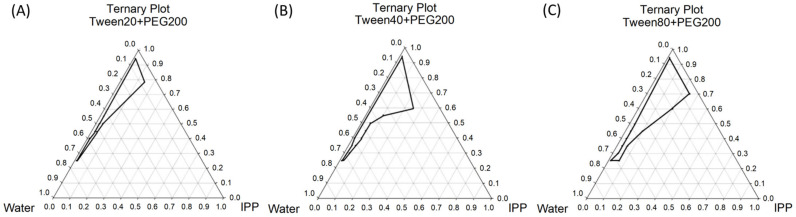
Pseudo-ternary phase diagram of the developed microemulsions: (**A**) ME-20-dA; (**B**) ME-40-dA; (**C**) ME-80-dA.

All the tested microemulsions were analyzed for transparency with a UV–vis spectrophotometer at a wavelength of 600 nm. If the optical density 600 (O.D. 600) value was lower than 0.2, the microemulsion was considered to have formed successfully. A microemulsion formulation with a relatively high ratio of surfactant/co-surfactant may have the chance to form a cubic phase or liquid crystalline structure. These structures can be confirmed by their non-flowable property when inverted 90° (increasing of viscosity) [23]. In this study, the tested formulations all retained fluidity, so we could confirm that the they were microemulsions. The results of this analysis are shown as pseudo-ternary phase diagrams in Figure 2. Among the three microemulsions, ME-40-dA and ME-80-dA have a larger microemulsion area than ME-20-dA, although the areas were only slightly different between the emulsions (Figure 2). Moreover, according to the results of the pseudo-ternary phase diagram, a relatively lower IPP content and higher water and surfactant levels were adequate for forming a microemulsion. This result is related to the O/W type of microemulsion, which is also in accordance with our purpose of encapsulating dA in the oil phase.

The surfactants used in this study were polysorbate-series surfactants. The hydrophilic head, polyoxyethylene, was the same in all surfactants, and their hydrophobic tails had differing carbon chain lengths (Figure 1). Among these, Tween 20 had a shorter hydrophobic tail and the highest hydrophilic–hydrophobic balance (HLB) value of 16.7. Tween 80 had the longest hydrophobic tail, so it had the smallest HLB value of 15. Between Tween 20 and Tween 80, Tween 40 had a medium HLB value of 15.6. Earlier studies established that an increase in the carbon chain lengths of a surfactant may decrease the critical micelle concentration (CMC) on the surface [24]. Therefore, for the O/W type of microemulsion, Tween 80, which had the longest hydrophobic tail and the lowest HLB value in this study, may have the advantage of forming micelles within a microemulsion system.

### 2.2. Stability Analysis of the Microemulsions Encapsulating dA

**Figure 3:**
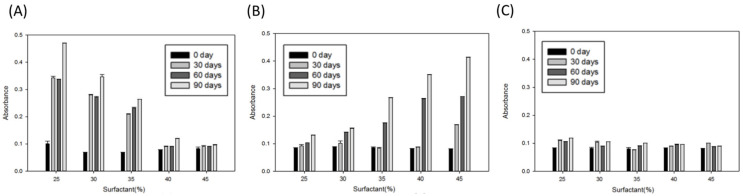
The visible light absorbance (O.D. 600) of microemulsions after different storage periods: (**A**) ME-20-dA; (**B**) ME-40-dA; (**C**) ME-80-dA. Data are presented as means ± SE (*n* = 3).

To conform the stabilities of the developed microemulsions at room temperature, we tested both the absorbance (at a wavelength of 600 nm) and the particle size at different storage periods from 0 to 90 days. The results are shown in Figure 2 and Figure 3, respectively. For absorbance, ME-80-dA had the most stable results for every surfactant ratio, at all storage time points. The absorbance values were all around 0.9 to 1.1, even when microemulsions were stored for up to 90 days (Figure 3C). However, compared with ME-80-dA, the absorbance of ME-20-dA and ME-40-dA varied among several surfactant ratios for storage times above 30 days. As shown by the results for ME-20-dA in Figure 3A, only the surfactant ratios of 40% and 45% had stable absorbances. Moreover, for ME-40-dA, lower surfactants ratios, 25% and 30%, had more stable absorbances (Figure 3B).

**Figure 4:**
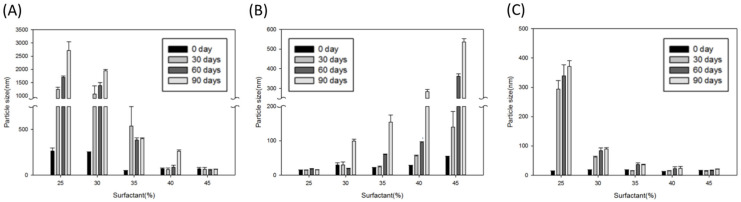
The particle size of microemulsions after different storage periods: (**A**) ME-20-dA; (**B**) ME-40-dA; (**C**) ME-80-dA. Data are presented as means ± SE (*n* = 3).

The results of particle size analysis of all microemulsions (Figure 4) almost correlated with the results of absorbance (O.D. 600) in Figure 3. For ME-20-dA and ME-40-dA, the particle sizes were obviously augmented when the absorbance increased. Only the ME-20-dA at the 45% surfactant ratio and the ME-40-dA at the 25% surfactant ratio kept a particle size lower than 30 nm after 90 days of storage (Figure 4A,B). Conversely, the particle sizes of ME-80-dA were all stable at ratios of 35%, 40% and 45%: the particle sizes ranged from 10 to 30 nm at every storage time point. However, at lower surfactant ratios, especially 25%, the particle sizes markedly increased after 30 days of storage. The largest particle size was 378 ± 19 nm at the 90-day time point (Figure 4C). This result was different from that of the absorbance analysis, which showed that the O.D. 600 values of ME-80-dA were quite stable at the 25% surfactant ratio. The difference might have been caused by ME-80-dA, with the 25% surfactant ratio, beginning the early-stage processes of instability, such as flocculation or coalescence, after 30 days. Accordingly, the enlarged particle sizes may not have immediately been reflected in the absorbance analysis results. The dilution of the sample used for particle size measurement may have influenced the micelles in the system. However, most formed microemulsion micelles are thermodynamically stable dispersions with ultralow interfacial tension. Therefore, the micelles of microemulsion can keep their particle structure in the proper dilution solutions. In addition, the results of particle size measurements to evaluate microemulsion formulation stability for this study still provide the constancy of particle size within each microemulsion formulation.

### 2.3. Cytotoxicity Analysis of the Microemulsions Encapsulating dA

**Figure 5:**
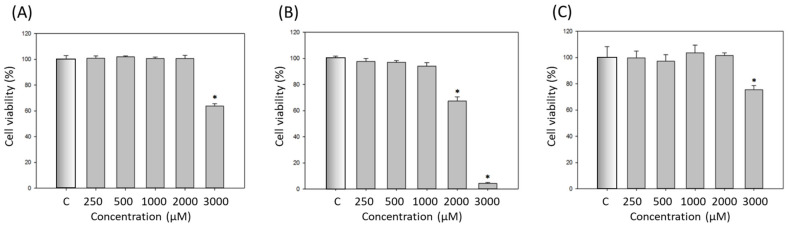
Cell viability of the polysorbate surfactants on B16-F10 cells: (**A**) Tween 20; (**B**) Tween 40; (**C**) Tween 80. C: control group. Data are presented as means ± SE (*n* = 3); * indicates a *p* value of less than 0.05 when compared with the control.

To measure both the cytotoxicity and the efficacy of the developed dA-containing microemulsions, we chose B16-F10 mouse melanoma cells as the test cell line in the following experiments. Firstly, the surfactants were all analyzed for their cytotoxicity using the standard 3-(4,5-dimethythiazol-2-yl)-2,5-diphenyl tetrazolium bromide (MTT) assay. The results are shown in Figure 5. In these results, Tween 20 and Tween 80 had no evident cytotoxicity on B16-F10 cells with concentrations lower than 2000 μM (Figure 5A,C). Conversely, the cytotoxicity of Tween 40 was higher than that of the others: the cell viability decreased to 70% at 2000 μM. The cell viability remained at 100% for Tween 40 at 1000 μM (Figure 5B). Although the cell viability clearly reduced when the concentrations of the Tween series surfactants were higher than 2000 or 3000 μM, the cytotoxicity of Tween series surfactants was still relatively lower than that of other commonly used surfactants, such as Triton X-100. The Triton X-100 surfactant killed nearly all the C2C12 mouse myoblast cells at 1% (*v*/*v*, approximately 144.45 μM) [25]. A previous study demonstrated the cytotoxic effect of Tweens using erythrocytes; the results revealed that Tween 20 and 40 at concentrations of 100 μM at 37 °C showed 95.4% and 91.1% hemolysis, respectively, while Tween 80 had only 61.0% hemolysis under the same conditions [26]. Thus, it was assumed that Tween 80 was less cytotoxic than Tween 20 and 40. Furthermore, the lower cytotoxicity of Tween 80 should be associated with its low HLB value compared to other Tween surfactants. Earlier studies also suggested that the cytotoxicity of the different types of surfactants can be ranked as follows: cationic surfactants have higher cytotoxicity than anionic surfactants; anionic surfactants have almost equal cytotoxicity to amphoteric surfactants; and non-ionic surfactants have the lowest cytotoxicity [27,28].

**Figure 6:**
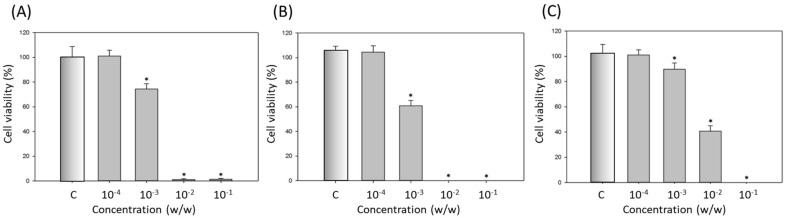
Cell viability of B16-F10 cells exposed to the microemulsions: (**A**) ME-20-dA; (**B**) ME-40-dA; (**C**) ME-80-dA. C: control group. Data are presented as means ± SE (*n* = 3); * indicates a *p* value of less than 0.05 when compared with the control.

The cytotoxicity of dA-containing microemulsions on B16-F10 cells are shown in Figure 6. Similar to the results in Figure 5, the cytotoxicity of ME-80-dA was relatively lower than that of other microemulsions, as the cell viability remained at 91 ± 1.8% when the dilution of ME-80-dA was 10^−3^ (*w*/*w*). The cell viability at the same concentration was only around 78% and 60% for ME-20-dA and ME-40-dA, respectively (Figure 6). Therefore, we can suggest that Tween 80 has a better safety profile than the other polysorbate surfactants both in the free surfactant condition and in the microemulsion. Furthermore, in the subsequent melanin content assay, we used the concentration of 10^−4^ (*w*/*w*) as the non-toxic condition to test the efficacy of dA-containing microemulsions.

The cytotoxicity of surfactants can be considered to be related to their amphiphilic structure, which can cause damage to cell membranes [29]. Moreover, a microemulsion could also cause cell membrane rupture not only through its composition but through the micelle structure [30]. Therefore, it is important for investigators to develop safe microemulsions to encapsulate the active compounds/drugs for use in human tissues, because they may otherwise bring about unexpected side effects and even toxicity. Consequently, we aimed to directly test the efficacy of microemulsions in a cell-based system, to avoid such cytotoxicity during treatment.

### 2.4. Efficacy of the Microemulsions Encapsulating dA

**Figure 7:**
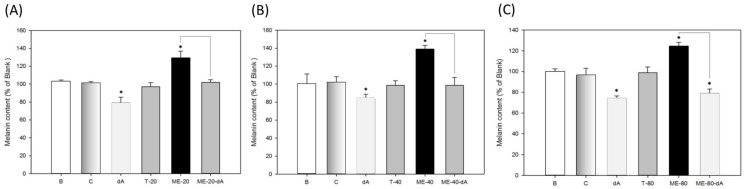
Melanin contents of B16-F10 cells treated with microemulsions: (**A**) ME-20-dA; (**B**) ME-40-dA; (**C**) ME-80-dA. B: blank group (medium only); C: control group (0.1% dimethyl sulfoxide as the solvent for dA); dA: deoxyArbutin (20 μM); T-20: Tween 20 (2000 μM); T-40: Tween 40 (1000 μM); T-80: Tween 80 (2000 μM); ME-20: Tween 20-based microemulsion (1 × 10^−4^ *w*/*w*); ME-40: Tween 40-based microemulsion (1 × 10^−4^ *w*/*w*); ME-80: Tween 80-based microemulsion (1 × 10^−4^ *w*/*w*); ME-20-dA: Tween 20-based microemulsion encapsulating dA (1 × 10^−4^ *w*/*w*); ME-40-dA: Tween 40-based microemulsion encapsulating dA (1 × 10^−4^ *w*/*w*); ME-80-dA: Tween 80-based microemulsion encapsulating dA (1 × 10^−4^ *w*/*w*). Data are presented as means ± SE (*n* = 3); * indicates a *p* value of less than 0.05 when compared with the control.

The encapsulated dA is a tyrosinase inhibitor that can suppress melanin production in melanocytes. Thus, to confirm its anti-melanogenesis activity, the melanin-producing B16-F10 cells were individually treated with the dA-containing microemulsions ME-20-dA, ME-40-dA and ME-80-dA for 24 h to evaluate their effects on melanin content. The results are shown in Figure 7. The melanin content of the blank group (medium only) is designated 100%; all other results are presented relative to the blank. For the control group (medium with solvent) and the free surfactants group (T-20, T-40 and T-80), the melanin content was almost the same as that of the blank group at 100%. For the dA group (free dA), the melanin content diminished to around 77 to 82% of the blank. This result also agreed with earlier studies showing that dA had the potent ability to inhibit the production of melanin in B16-F10 cells [3]. Besides this, to assess the effects of the dA-containing microemulsions, we used ME-20, ME-40 and ME-80 (Tween series surfactant-based microemulsions) to assess the variations in melanin content. Shown in Figure 7, the melanin content of the ME-20, ME-40 and ME-80 groups increased to around 130 to 140% of the blank. However, it was clear that the melanin contents of the ME-20-dA, ME-40-dA and ME-80-dA groups all recovered to at least 102% of blank, and the melanin content of ME-80-dA even reached 81% (Figure 7C). The increase in the melanin content of the ME-20, ME-40 and ME-80 groups was caused by the microemulsions promoting the destruction of the melanin-forming melanosome in melanocytes, which is the reason why the melanin content of surfactant-based microemulsions was higher than that of the blank and the control group; it reflects an increase in the extraction rate rather than an increase in the melanin yield.

The molecular weight of dA is 194.23 g/mol. In the melanin content assay, all dA-containing microemulsion groups had a final dA concentration of only 5.15 μM. Therefore, the dA concentration in the ME-20-dA, ME-40-dA and ME-80-dA formulations were lower than that of the free dA group (20 μM). Consequently, the effects of ME-20-dA, ME-40-dA and ME-80-dA on melanin production could be observed in a comparison of the melanin contents between surfactant-based microemulsions and dA-containing microemulsions: the melanin content of the ME-20-dA and ME-40-dA groups decreased to a basal level (Figure 7A,B). In addition, the ME-80-dA group had the lowest melanin content, and difference between the ME-80 and ME-80-dA groups was up to~50% of the blank (Figure 7C). Therefore, we can postulate that dA-containing microemulsions had a great ability to inhibit melanin production when we directly assessed this effect in B16-F10 cells.

## 3. Materials and Methods

### 3.1. Materials

DeoxyArbutin (dA) was acquired from Denjelly Co., Ltd. (Miaoli, Taiwan). The polysorbate surfactants, Tween 20, Tween 40 and Tween 80, were purchased from Sigma-Aldrich (St. Louis, MO, USA). Isopropyl palmitate (IPP), polyethylene glycol 200 (PEG 200), dimethyl sulfoxide (DMSO), sodium hydroxide (NaOH), sodium chloride (NaCl) and other chemicals were purchased from Sigma-Aldrich (St. Louis, MO, USA). Dulbecco’s modified Eagle medium (DMEM), fetal bovine serum (FBS), sodium pyruvate, penicillin, streptomycin, trypsin-EDTA and other cell culture agents were purchased from Gibco BRL/Invitrogen (Carlsbad, CA, USA). The 3-(4,5-dimethylthiazol-2-yl)-2,5-diphenyl tetrazolium bromide (MTT) was purchased from Affymetrix/USB (Cleveland, OH, USA). Deionized distilled water (ddH_2_O) for the microemulsions and buffers was produced by the Milli-Q system (Millipore, Bedford, MA, USA).

### 3.2. Microemulsion Preparation

All components of the microemulsions were weighed to obtain the intended proportion. Firstly, the Tween series surfactants were mixed with PEG 200 at a 4:1 ratio, then mixed with the dA-containing IPP at different ratios. Finally, the appropriate amount of water was added to 100%. If the emulsion could form spontaneously, the micelles would be small enough to be transparent.

### 3.3. Transparency Analysis and Pseudo-ternary Phase Diagram Drawing

For transparency analysis, all the tested samples were added to a quartz cuvette and then analyzed by a UV–vis spectrophotometer (T60, PG Instruments Ltd., Lutterworth, UK) at a wavelength of 600 nm. If the optical density 600 (O.D. 600) value was lower than 0.2, the microemulsion was considered to have been established successfully. Therefore, a pseudo-ternary phase diagram was drawn to visualize the microemulsion range.

### 3.4. Particle Size Analysis

For particle size analysis, the 1 μL samples were diluted 1000 times with ddH_2_O, then the prepared sample was put into a quartz cuvette and then analyzed by a particle size analyzer (Zetasizer Nano Series S, Malvern Panalytical Ltd., Worcestershire, UK).

### 3.5. Stability Analysis

To analyze the stability of the microemulsions, we stored all the designed samples at room temperature (around 25 °C) for 30 days, 60 days and 90 days. The samples were analyzed for the transparency and particle size to confirm whether these characteristics had changed.

### 3.6. Cell Lines and Cell Culture

The cell line B16-F10 (BCRC 60031) of mouse melanoma cells was purchased from the Bioresource Collection and Research Center (BCRC, Hsinchu, Taiwan). B16-F10 cells were cultured in DMEM supplemented with 10% FBS, 2 mM glutamine, 100 mg/mL streptomycin and 100 U/mL penicillin. Cells were kept in a humidified environment with 5% CO_2_ at 37 °C. Cells were sub-cultured for 3 to 4 days to maintain the normal growth conditions and monitored with an inverted microscope (CKX41SF, Olympus, Tokyo, Japan) to ensure they kept an ordinary morphology.

### 3.7. MTT Analysis

For MTT analysis, according to an earlier study [31], the cells were seeded in 96-well plates at a density of 8 × 10^3^ cells/well and cultured for 24 h. The medium was replaced with fresh medium and the cells were treated with samples at different concentrations for another 24 h. Next, 100 μL of an MTT solution (0.5 mg/mL) was added to the wells then incubated for 30 min at 37 °C. After washing twice with phosphate-buffered saline (PBS), the tested cells were directly lysed with 100 μL of DMSO. Finally, the absorbance was measured at 540 nm with an ELISA microplate reader (BioTek, Seattle, WA, USA). The cell viability was presented as a percentage of cell viability in untreated control cells.

### 3.8. Melanin Content Analysis

For melanin content analysis, according to an earlier study [32], the B16-F10 cells were pre-cultured in 6-well plates for 24 h at a density of 8 × 10^4^ cells/well, then the cells were treated with different samples for an additional 24 h. After washing steps, the treated cells were lysed in 120 μL of NaOH solution (1 N) at 65 °C for 1 h to dissolve the melanin molecules into the solution. The total melanin contents in the solutions were examined at 405 nm with the ELISA microplate reader. The melanin content was calculated as a percentage of melanin content in the untreated control cells. 

### 3.9. Statistical Analysis

The experimental results were investigated by Student’s *t*-tests and are all shown as means ± standard errors (S.E.) of three independent experiments. The *p*-values were also calculated, and those less than 0.05 were considered as significant.

## 4. Conclusions

**Figure 8:**
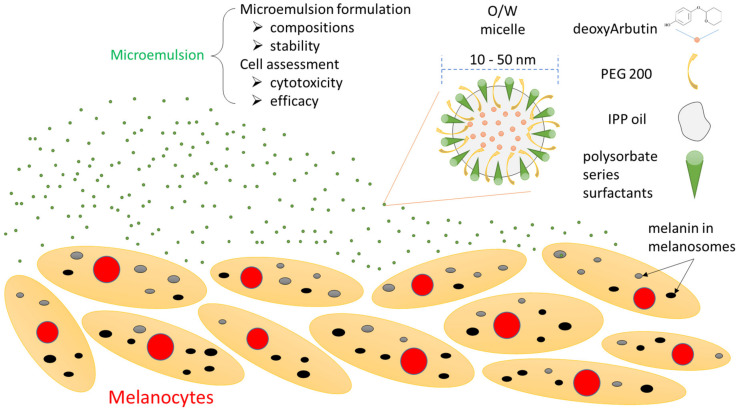
Summary of the cell-based system used to directly assess the effects of the microemulsions encapsulating deoxyArbutin.

In conclusion, the present study achieved its aim of direct evaluation of microemulsion in cells, as summarized in Figure 8. The relative safe/non-toxic O/W microemulsions encapsulating dA were successfully formulated from polysorbate-series surfactants, the co-surfactant, PEG 200 and IPP oil. The transparency and particle size at different storage time periods revealed that the developed microemulsions were stable for a long storage time. Most importantly, the effects of the diluted microemulsion samples were directly assessed in B16-F10 cells, revealing that the anti-melanogenesis activity of dA-containing microemulsions was markedly better than that of the free dA group. Among them, ME-80-dA had the best ability to suppress melanin production, achieving approximately 50% of the blank. Therefore, our results demonstrated that this microemulsion encapsulating deoxyArbutin may allow the use of deoxyArbutin instead of hydroquinone to treat dermal hyperpigmentation disorders in the future.

## Figures and Tables

**Table 1:** Compositions of the deoxyArbutin-containing microemulsions.

	Tween 20/PEG 200 (4:1)	Tween 40/PEG 200 (4:1)	Tween 80/PEG 200 (4:1)	dA	IPP	ddH_2_O
ME-20-dA	25−45	-	-	1	2	52−72
ME-40-dA	-	25−45	-	1	2	52−72
ME-80-dA	-	-	25−45	1	2	52−72
All values are presented in % (*w*/*w*)						

## Data Availability

The data presented in this study are available for research purposes on request from the corresponding author.
